# Developing entrustable professional activities for family medicine training in South Africa

**DOI:** 10.4102/safp.v65i1.5690

**Published:** 2023-02-17

**Authors:** Louis S. Jenkins, Robert Mash, Ts’epo Motsohi, Mergan Naidoo, Tasleem Ras, Richard Cooke, Hanneke Brits

**Affiliations:** 1 Department of Family and Emergency Medicine, Faculty of Medicine and Health Sciences, Stellenbosch University, Cape Town, South Africa; 2 Primary Health Care Directorate, Department of Family, Community and Emergency Care, Faculty of Health Sciences, University of Cape Town, Cape Town, South Africa; 3 Department of Family and Emergency Medicine, George Hospital, Western Cape Department of Health, George, South Africa; 4 Discipline of Family Medicine, School of Nursing and Public Health, College of Health Sciences, University of KwaZulu-Natal, Durban, South Africa; 5 Division of Family Medicine, Department of Family, Community and Emergency Care, Faculty of Health Sciences, University of Cape Town, Cape Town, South Africa; 6 Department of Family Medicine and Primary Care, Faculty of Health Sciences, University of the Witwatersrand, Johannesburg, South Africa; 7 Department of Family Medicine, Faculty of Health Sciences, University of the Free State, Bloemfontein, South Africa

**Keywords:** entrustable professional activities (EPAs), family medicine, specialist, training, South Africa

## Abstract

Workplace-based assessment (WPBA) is becoming part of high-stake assessments in specialist training. Entrustable professional activities (EPAs) are a recent addition to WPBA. This is the first South African publication on developing EPAs for postgraduate family medicine training. An EPA is a unit of practice, observable in the workplace, constituting several tasks with underlying knowledge, skills and professional behaviours. Entrustable professional activities allow for entrustable decisions regarding competence in a described work context. A national workgroup representing all nine postgraduate training programmes in South Africa has developed 19 EPAs. This new concept needs change management to understand the theory and practice of EPAs. Family medicine departments with large clinical workloads are small, necessitating navigating logistical issues to develop EPAs. It has unmasked existing workplace learning and assessment challenges.

**Contribution:**

This article contributes new thinking to developing EPAs for family medicine in an effort to understand more authentic WPBA nationally.

## Introduction

Workplace-based assessment (WPBA) is becoming more important globally and in South Africa as a part of high-stake assessment decisions in training medical specialists.^1^ Increasing awareness of modern medicine’s failure to meet society’s health needs, with concomitant concerns around professionalism, has prompted reviews of assessment methods.^2,3^ Part of the problem is the challenges with assessing the performance of medical professionals in an authentic and entrustable way. Subsequently, there has been a shift from predominantly written and clinical summative examinations towards outcomes-based education and, more recently, competency-based medical education.^4,5^ This model ensures that the public and the professions can trust that a medical professional is competent to practice. The need to observe and document workplace assessment of actual professional activities using a structured format became apparent around 2005.^6^ Assessing workplace-based professional activities in a trustworthy and authentic way within gave rise to entrustable professional activities (EPAs) as a novel method of competency-based assessment.

Entrustable professional activities are a relatively recent addition to WPBA in health professions education at both under- and post-graduate levels.^6,7^ They do not replace curricula or competency frameworks but are informed by them. They provide the ‘bridge’ between curricula learning outcomes, competency frameworks and everyday professional activities in the clinical workplace.^8^ The ‘EPAs are units of professional practice that capture essential competencies in which trainees must become proficient before undertaking them independently’.^9^ They provide supervisors with evidence that trainees can perform tasks independently and may align to more than one learning outcome.

The concept of ‘trust’ is integral to EPAs.^10^ Clinical trainers and supervisors need to trust that learners, in this case, registrars in postgraduate family medicine training, have developed the range of knowledge, skills, attitudes and professional behaviours expected from a family physician. This is even more true for the public, who must use and trust the health system and the healthcare provider.^11^ Ultimately, we must trust that our new specialists can deliver the appropriate activities in the workplace. It becomes more authentic to define these activities and assess them rather than more abstract educational concepts found in competency frameworks (e.g. communicator, scholar) or learning outcomes.^12^ Supervisors make ad hoc entrustment decisions every day when registrars are allowed to engage with patients and boundaries are set regarding the amount of supervision required. With EPAs, these entrustment decisions are more clearly defined and formalised.

**FIGURE 1:**
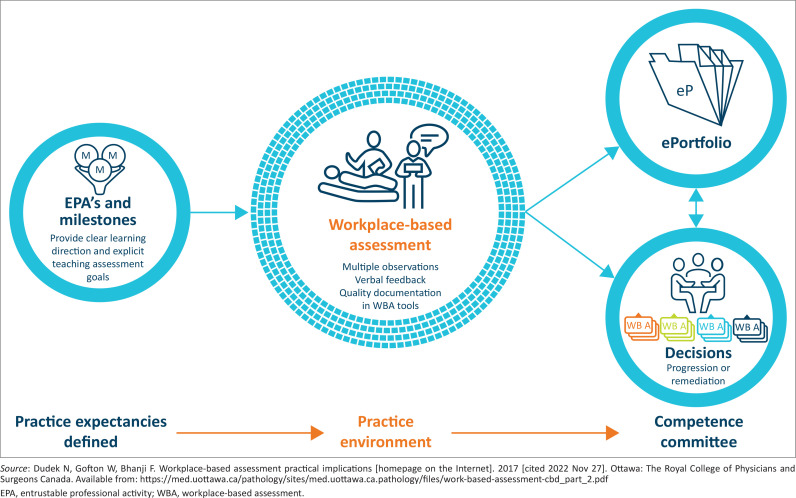
Key components of workplace-based assessment.

Many developed countries have published EPAs. The Association of Faculties of Medicine of Canada developed 35 EPAs for family medicine,^13^ while the American Colleges of Graduate Medical Education has developed 76 EPAs for family medicine.^14^ Literature on the use of EPAs in sub-Saharan African countries could not be found. Several postgraduate fellowships, masters and postgraduate diploma programmes in South Africa have begun writing EPAs. An example is the national Diploma in Human Immunodeficiency Virus (HIV) Medicine for the Colleges of Medicine of South Africa (CMSA). The South African Committee of Medical Deans have committed to incorporating WPBA in all postgraduate training programmes. The CMSA has indicated that WPBA should be part of licencing examinations for all medical specialities by 2024. This involves four work streams: curricular redesign, EPA development, utilisation of appropriate technology and operationalisation of WPBA. Entrustable professional activities form part of the four key components of WPBA. Assessments are ultimately captured in a portfolio. These assist competence committees in making summative decisions on registrar progression or licencing as a family physician. The four key components are illustrated in Figure 1^15^:

- A clear description of what must be achieved by the end of the training programme. Programmatic learning outcomes and competency frameworks have traditionally defined these as individual outcomes; however, EPAs tend towards integrating learning outcomes and may be contextually appropriate.
- A gathering of information through WPBA to provide evidence on what the registrar does in the workplace environment. Each observation or judgement of the registrar is called a ‘data point’. Effective WPBA requires multiple data points by numerous assessors, using various low-stake assessments, in different clinical contexts, throughout the training programme. Although each point is not a reliable assessment, they can provide a reliable evaluation when aggregated together to inform high-stake entrustment decisions around EPAs.^10^
- The portfolio provides a way of collecting and presenting the information gathered. These portfolios have more utility when they are electronic. E-portfolios are easier to structure, contribute to from a distance, provide quality assurance and are used in national assessments.^16^
- A way of making a final high-stake assessment based on the evidence presented in the portfolio. A competence committee uses the portfolio to assess the registrar’s competence independently. The respective Colleges in South Africa would probably establish their discipline-specific committees.

This article aims to create awareness of the EPA discourse in South Africa, particularly in family medicine. The objectives include updating colleagues on progress made, stimulating thinking to help solve complex concepts and facilitating buy-in towards implementing more authentic assessments.

## What is an entrustable professional activity

An EPA is a unit of practice, observable in the clinical workplace and constituting several tasks with underlying knowledge, skills, attitudes and professional behaviours.^17^ A unit of practice is seen as a delineated part of the work expected in that context and for which a decision must be made on the level of trust a person is given to perform that work. The level of trust can be graded on a scale as follows^17^:

- The registrar can observe only, but not enact the EPA
- The registrar is allowed to perform the EPA with direct and proactive supervision in the room
- The registrar is allowed to perform the EPA, but supervision is readily available if needed
- The registrar is allowed to perform the EPA unsupervised
- The registrar is allowed to provide supervision to more junior learners

For example, a unit of practice might mean being the doctor responsible for the labour ward or the doctor responsible for the patient list in the operating theatre. Assessing a professional task in the workplace and making an entrustment decision on the level of competency of the registrar constitutes an EPA.^6^ The constituent tasks of an EPA and the underlying knowledge, skills, attitudes and professional behaviours will touch on multiple educational learning outcomes.

Once you have defined the unit of practice, you must then elaborate on the details of the EPA. The Association of Medical Education in Europe Guide 140 offers a standard template for EPA writing, constituting eight headings.^17^ The EPA template typically includes the following:

- The title of the EPA
- Specifications of what is included in the activity and what is not
- The potential risks for patients if the activity is performed poorly
- How the EPA links to the national educational unit standards
- The required knowledge, skills, attitudes and experience
- Sources of evidence to support an entrustment decision by a competency committee
- Level of entrustment that should be achieved
- When this EPA must be re-assessed if not practiced for a time.

The conceptualisation of the work of family physicians in the workplace in South Africa has broadly defined three key roles. These roles are clinician and consultant, capacity builder and clinical trainer and leader of clinical governance.^18^ The workplace can be primary healthcare, the district hospital or district clinical specialist teams. These broad roles need to be unpacked into the units of practice. In postgraduate family medicine training, we have not adopted a competency framework like the popular CanMeds framework.^19^ However, we have defined programmatic learning outcomes and categorised them into five national unit standards.^20^ These unit standards broadly focus on learning outcomes for:

- Leadership and clinical governance
- Clinical practice
- A community-orientated model of primary healthcare
- Clinical teaching and training in the workplace
- Professionalism and ethical behaviour

Therefore, linking our EPAs to these unit standards rather than a competency framework in our context makes sense. Typical training programmes have 5–10 EPAs per course year.^7^ For the 4-year family medicine programme, this would amount to about 30–40 EPAs. Training programmes will evaluate progress against the EPAs across the programme’s lifespan and embed these ‘medium-stake’ assessments into their approach to programmatic assessment. The competency committee at the College of Family Physicians of South Africa (SA) will then use the same portfolio to make a final ‘high-stake’ assessment at the end of training.

## Progress to date

The College of Family Physicians of SA started exploring EPAs to train family physicians in 2020. While postgraduate exit level assessment falls within the ambit of the College of Family Physicians, the writing, blueprinting and implementing of EPAs for workplace-based training and assessment is a function of the Education and Training Committee of the South African Academy of Family Physicians (SAAFPs). In practice, because of the small number of faculty members among the nine postgraduate training programmes in the country, there is considerable overlap between the members of these two structures.

A national working group was established with representatives from all nine postgraduate programmes. These representatives were opportunistically recruited through volunteers who committed to representing their university training programme. The national EPA working group met several times from 2020 to 2022 via virtual meetings, learning how to develop EPAs together. The chairperson of the working group underwent the online international training course offered by the University Medical Centre Utrecht in the Netherlands, under the directorship of Prof Olle ten Cate. Nineteen draft EPAs have been written, discussed, edited and refined (see Table 1). This is a work in progress. Face-to-face workshops and online collaborative meetings are planned for 2023, with a list of nationally agreed EPA titles finalised by mid-2023 and the ratified EPAs confirmed by the end of 2023. Implementation, user buy-in and incorporation into the existing learning portfolio will be ongoing in 2024.

**TABLE 1:** Draft entrustable professional activity titles for postgraduate family medicine training in South Africa.

No.	EPA title
1.	Managing adults and children with palliative care needs
2.	Managing adult patients with orthopaedic conditions
3.	Managing common ear, nose and throat emergency presentations
4.	Conducting a consultation for mental health disorders
5.	Developing a management plan for a mental health user regarding psychopharmacological agents
6.	Managing an adult patient with a common medical condition
7.	Managing an adult patient with an urgent or emergency condition
8.	Managing an adult patient with an undifferentiated condition
9.	Managing women with health-related procedures
10.	Managing district level adult patients with HIV and TB
11.	Managing common emergencies in infants and children
12.	Managing common conditions of infants and children
13.	Managing common and emergencies conditions in neonates
14.	Administer general anaesthesia for district-level adult patients
15.	Administer spinal anaesthesia for district-level adult patients, including pregnant women
16.	Administer local anaesthetics and regional blocks for district-level adult patients
17.	Managing patients with surgical problems
18.	Managing patients presenting with sexual, physical or emotional assault
19.	Leading clinical governance activities

HIV, human immunodeficiency virus; TB, tuberculosis.

As these draft EPAs have been revisited and rediscussed during the workshops and meetings, it has become clear that many of them need further editing, simplifying and combining as a smaller set of units of practice in the workplace, as defined by each EPA.

An EPA workshop was held at the 24th National Family Practitioners’ Conference in Cape Town in August 2022. Twenty people from various universities, including the neighbouring Southern African Development Community, contributed. The workshop provided an opportunity to review and engage with the EPA writing process to date. It also highlighted some challenges and transversal principles for EPA writing. Subsequently, two more national 1-day workshops were held in October 2022, one in Gauteng hosted by the University of Witwatersrand and one in Cape Town hosted by Stellenbosch University. The main areas discussed included developing EPAs for our discipline, programmatic assessment and using a national e-portfolio. Experts in health professions education from Maastricht and Utrecht universities in the Netherlands provided a global perspective.

## Discussion

### Change management and logistical issues

**FIGURE 2:**
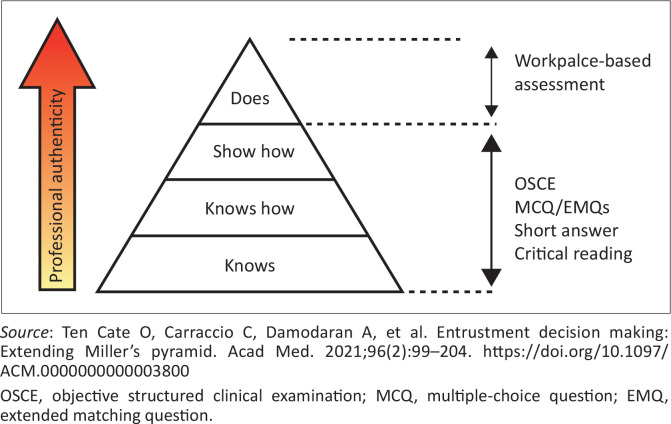
Design of the national licencing examination and Miller’s pyramid.

The concept of EPAs is very new to most clinicians and faculty members. Furthermore, the nine family medicine departments have few full-time academic staff and a heavy clinical workload. Getting representatives from all training programmes on the national working group and then having regular meetings, with ongoing training and work developing EPAs, are big challenges. On the positive side, all nine programmes agree that WPBA to measure EPAs is much needed to improve the authenticity of our assessments. Currently, the national examination can only reliably assess knowledge, the application of knowledge and performance under simulated and restricted conditions, such as an objective structured clinical examination (Figure 2). Workplace-based assessment provides a reliable means of assessing the full range of workplace activities in a reliable way. The well-known Miller’s pyramid can be expanded to include ‘Trust’ (with future care) above ‘Does’.^12^

### Understanding the theory and practice of entrustable professional activities relevant to family medicine

From the literature^1,6,7,8,10^ and the three workshops, the following lessons have been learnt:

- In deciding on the most appropriate titles for EPAs, we have moved from writing EPAs for specific clinical skills towards less granular EPAs. For example, whether the registrar can be trusted to manage a primary health care (PHC) clinic or a district hospital theatre list. This will help to have a manageable number of EPAs (about 30–40 for the 4-year programme) and align better with actual practice units. Multiple assessors must observe and assess all EPAs in the workplace over time to ensure sustainability. Because of the wide range of skills expected of a family physician, it is essential to limit the number of EPAs. This requires a conceptual shift to focus on the workplace rather than the curriculum, competency framework or learning outcomes.
- Make the clinical context explicit for each EPA. For example, does it refer to primary healthcare (including the community and the facility) or the district hospital? The national curriculum for family medicine training covers the whole district health ecosystem.
- The EPAs can refer to external sources rather than repeating information multiple times. For example, a list of clinical conditions and procedures will be defined in the curriculum. Writing the EPAs also presents the opportunity to update the national curriculum.
- Some aspects are transversal across EPAs, such as person-centredness and professional and ethical behaviour. The EPA guideline defines the general features of learners that enable trust and can be remembered with the acronym ‘A RICH’ assessment^21^ (Table 2).
- Defining the sources of evidence for entrustment decisions helps to design the portfolio and how the contents are aggregated to assess the EPAs.
- It is not necessary to assess all the specifications of an EPA. For example, neither every skill nor the management of every possible condition and complication need to be assessed. The registrar should show evidence of adaptive competence. In other words, based on the evidence, can they be trusted to handle new challenges in this unit of practice in the future?^10^ The principle is that we are assessing colleagues for tomorrow’s (not just present) entrustability.
- Entrustment levels must be agreed upon nationally, achievement of which must consider the professional behaviours of the registrar, the complexity of the EPA, the professional working relationship between the supervisor and the registrar and the context.^17^ For example, level 2 at the end of year one (proactive supervision), level 3 (reactive supervision) at the end of year two and level 4 by the end of the programme, which would mean being able to work independently. Importantly, a summative decision on entrustment is taken by a committee, not an individual.
- Moving from a time-based mindset to an entrustment-based mindset will take commitment by both faculty and registrar. The change management is significant; faculty development and communication are critical.

One of the challenges for more resource-constrained settings may be the alignment of the entrustment decision for the EPA with the reality in the workplace. In a learning environment with insufficient supervision and high service demands, it is often necessary to entrust people to deliver services for which they may not be fully prepared. The actual level of entrustment may therefore precede the assessed level of entrustment and implied level of supervision. This paradoxical challenge poses an opportunity for innovative re-thinking of standardising and benchmarking entrustment decisions across our training landscape and how to ensure we train people in appropriate facilities and learning environments.

**TABLE 2:** Professional behaviour that enables trust.

Feature	Definition
Agency	Proactive attitude towards work, team, safety and personal development that includes awareness of and responding to the need for action, even when outside of the strict definition of one’s responsibilities and practice of adaptive expertise.
Reliability	Consistent, predictable and conscientious behaviour, driven by a sense of accountability and responsibility
Integrity	Truthfulness, benevolence and person-centredness, where expertise is employed to benefit patients and decisions are motivated by concern for and made in the best interests of patients (and their families).
Capability	The ability to perform a specific task in a variety of contexts and within an appropriate time frame, requiring a reasonable understanding and overall view of the clinical situation and ability to communicate and work effectively with others within a system
Humility	Discernment of one’s limitations; willingness and ability to ask for help and feedback; receptivity to insights of patients and co-workers and ability to learn and develop from mistakes, feedback and the expertise of others

*Source:* Ten Cate O, Carraccio C, Damodaran A, et al. Entrustment decision making: Extending Miller’s pyramid. Acad Med. 2021;96(2):99–204. https://doi.org/10.1097/ACM.0000000000003800

### Unmasking workplace learning and assessment challenges

The development of EPAs as one of the critical components of WPBA has also exposed the need for work in other areas. Currently, it is clear that the training programmes do not fully trust the reliability of WPBA and that we are not yet ready to include it in high-stake assessments. One of the workshops explored the issues that need to be addressed to enable us to trust WPBA. These included:

- Ensure judgements are made by assessors who understand the EPAs and requirements of the training programmes. Many programmes rely heavily on specialists from other disciplines in regional or tertiary hospitals who do not fully understand family medicine training programmes.
- Increase the number of different assessors providing data points. This may require innovation to include more family physicians using indirect assessments and other suitable assessors such as senior medical officers, senior registrars, other specialists, senior nurses and even patients.
- Train assessors to understand the EPAs and WPBA. In addition, we need to benchmark the scoring of data points against an agreed standard and ensure meaningful written feedback is also included. Writing constructive feedback and not just scoring is a challenge that is important for learners and needed by competency committees.
- Ensure that data points come from both PHC and district hospitals, as the PHC level tends to be neglected in assessments.
- Agree on critical minimum standards for training programmes and accrediting the competency of clinical trainers may be valuable.
- Use cost-effective technology to facilitate WPBA and assist with entrustment decisions.

In addition, developing an e-portfolio is essential, and almost all postgraduate family medicine training programmes in South Africa have now adopted the SCORION^©^ platform that the SAAFP is coordinating. Key issues that remain with the e-portfolio include:

- Supporting implementation across all training programmes, fixing minor glitches and developing a management dashboard.
- Planning a significant architecture revision to include the EPAs and aggregate data points to assess them. Currently, the portfolio rewards completion of the portfolio more than progress towards the unit standards or, in future, EPAs.

Finally, we will need to develop a national competency committee structure to make high-stake decisions. This will most likely be situated within the College of Family Physicians and will initially focus on ensuring that all programmes are performing WPBA to an acceptable standard and providing quality assurance and feedback on the e-portfolios. While doing this, they can develop capacity and processes for assessing the e-portfolios and making a summative judgement by 2024. These processes must also be aligned with the national drive to implement WPBA in all clinical disciplines.

## Conclusion

Developing and writing EPAs for family medicine is a helpful and stimulating discursive journey. It is part of a more significant journey towards using WPBA in programmatic assessment and the national licensing examination. It has exposed the variability between training programmes and the need to collaborate on EPA development, further curriculum review, improve clinical training, implement the e-portfolio and design a process for high-stake decision-making in the College of Family Physicians.
